# Does Exercise Influence the Susceptibility to Arterial Thrombosis? An Integrative Perspective

**DOI:** 10.3389/fphys.2021.636027

**Published:** 2021-02-23

**Authors:** Line Nørregaard Olsen, Mads Fischer, Phillip Adrian Evans, Lasse Gliemann, Ylva Hellsten

**Affiliations:** ^1^Department of Nutrition, Exercise and Sports, University of Copenhagen, Copenhagen, Denmark; ^2^Haemostasis Biomedical Research Unit, Welsh Centre for Emergency Medicine Research, Morriston Hospital, SBU Health Board, Swansea, United Kingdom; ^3^College of Medicine, Swansea University, Swansea, United Kingdom

**Keywords:** physical activity, exercise, thrombogenicity, blood clots, platelet reactivity, clot microstructure, plasma biomarkers

## Abstract

Arterial thrombosis is the primary cause of death worldwide, with the most important risk factors being smoking, unhealthy diet, and physical inactivity. However, although there are clear indications in the literature of beneficial effects of physical activity in lowering the risk of cardiovascular events, exercise can be considered a double-edged sword in that physical exertion can induce an immediate pro-thrombotic environment. Epidemiological studies show an increased risk of cardiovascular events after acute exercise, a risk, which appear to be particularly apparent in individuals with lifestyle-related disease. Factors that cause the increased susceptibility to arterial thrombosis with exercise are both chemical and mechanical in nature and include circulating catecholamines and vascular shear stress. Exercise intensity plays a marked role on such parameters, and evidence in the literature accordingly points at a greater susceptibility to thrombus formation at high compared to light and moderate intensity exercise. Of importance is, however, that the susceptibility to arterial thrombosis appears to be lower in exercise-conditioned individuals compared to sedentary individuals. There is currently limited data on the role of acute and chronic exercise on the susceptibility to arterial thrombosis, and many studies include incomplete assessments of thrombogenic clotting profile. Thus, further studies on the role of exercise, involving valid biomarkers, are clearly warranted.

## Introduction

Thrombosis is the formation of a blood clot inside a blood vessel, leading to obstruction of blood flow in the arterial or venous circulatory system (Mackman, 2008). Acute arterial thrombosis is the cause of myocardial infarction and stroke, which collectively are the most common causes of death in developed countries (Mozaffarian et al., 2016). The risk of thrombosis and the consequent cardiovascular events are closely coupled to aging and lifestyle factors such as a diet, smoking, and physical inactivity.

A primary trigger of arterial thrombosis is rupture of an atherosclerotic plaque, which leads to a rapid recruitment of platelets to the injured site, through interaction of platelet surface receptors with collagen and other proteins exposed at the site of injury in the vessel wall. Subsequent activation of the coagulation cascade, leading to generation of thrombin and fibrin, results in clot formation and occlusion of the artery (Mackman, 2008). The ultimate microstructure of the formed blood clot is of significance; less dense clots can be dissolved naturally through the process of fibrinolysis, whereas more dense blood clots cannot easily be dissolved, and consequently, dense blood clots are associated with higher risk of thromboembolic events (Collet et al., 2000; Mills et al., 2002). Thus, the severity of a blood clot is closely associated with the structure of the fibrin network, which is supported by the finding that the rate of fibrinolysis is dependent on the structure formed by the fibrin fibers (Collet et al., 2006). Therefore, the potential for thrombosis formation, the density of the thrombus, and the efficacy of fibrinolysis are of clinical importance in evaluating the risk of arterial thrombosis.

Lifestyle changes, such as regular physical activity, have protective effects on cardiovascular disease, such as acute myocardial infarction and stroke (Buckley et al., 2019) and habitual physical activity is accordingly recommended for populations at risk (Arnett et al., 2019; Pelliccia et al., 2020). Nevertheless, there is a risk-benefit paradox with regard to physical activity; acute exercise influences the coagulation system with a consequent transient increase in the risk of arterial thrombosis, with the magnitude of risk being influenced by physical fitness and relative exercise intensity (Thompson et al., 2007, 2020). This brief review covers some of the known effects of acute and habitual exercise on risk markers of arterial thrombosis. We first provide a short overview over experimental methods used to assess the integrated risk of arterial thrombosis, followed by a discussion on the influence of acute exercise and exercise training on biomarkers of arterial thrombosis.

## Biomarkers Indicating the Susceptibility to Arterial Thrombosis

### Platelet Reactivity

Platelets play an important role for many physiological and pathophysiological processes including healing of injured blood vessels and the development of arterial thrombosis (Nachman and Rafii, 2008; de Groot et al., 2012). Platelets circulate with the blood in an inactivated state until they come into contact with activating molecules, such as circulating epinephrine and collagen exposed upon injury to the vessel wall. The activated platelets adhere and initiate a tightly regulated process leading to the formation of a hemostatic plug (de Groot et al., 2012).

Evaluation of platelet aggregation can include platelet number, morphology, and function (Vinholt et al., 2014) and there are several available methods for these assessments as described in detail elsewhere (Vinholt et al., 2014). The present review only focuses on the assessment of platelet reactivity. For this assessment, light transmission aggregometry is often used (Born, 1962; O’brien, 1962; Vinholt et al., 2017), and reactivity is determined by assessing the propensity of platelets to aggregate in response to different concentrations of platelet receptor agonists (Algahtani and Heptinstall, 2017). Physiologically relevant agonists for the assay are collagen, adenosine diphosphate (ADP), and thrombin receptor-activating peptide (TRAP) (Born, 1962; Davidson et al., 1988; Lundberg Slingsby et al., 2017; Vinholt et al., 2017).

Although light transmission aggregometry is a useful method for the assessment of platelet function, a few aspects are useful to keep in mind when interpreting the data. One limitation of assessing platelet function *per se* as a marker of blood clot susceptibility is that it only provides indication of one, albeit important, step in hemostasis. Methodological limitations include preparation time of platelet rich plasma to be used in the assay, which may influence aggregation, and that the measurement is made *in vitro*, without immediate influence of hemodynamic factors. Nevertheless, with careful methodological considerations and in combination with other markers of thrombogenicity, the platelet aggregometry method provides a useful indication of the susceptibility to thrombosis, and the method also has the advantage that a large number of samples, and many different agonists, can be tested simultaneously (Salehi et al., 2014; Paniccia et al., 2015; Vinholt et al., 2017).

### Plasma Markers of Thrombogenicity

Various markers of hemostasis and fibrinolysis have been identified as independent cardiovascular risk factors (Koenig and Ernst, 2000) and have been widely used in clinical practice. Standard coagulation assays include assessment of activated partial thromboplastin time (APTT), prothrombin time (PT), and thrombin time (TT) (Feng et al., 2014). The assays are functional and evaluate the rate of clot formation when the coagulation cascade has been activated. Other commonly used markers of thrombogenicity are related to fibrin, which plays an essential role in the microstructure of the blood clot. Blood clots can be dissolved by the fibrinolytic system through degradation of fibrin. The key enzyme for degradation of fibrin is plasmin, which is converted from circulating plasminogen by plasminogen activators: tissue-plasminogen activator (t-PA) and urokinase-plasminogen activator (u-PA). The t-PA and u-PA can be inhibited by plasminogen-activator inhibitor-1 (PAI-1) and plasminogen-activator inhibitor-2 (PAI-2).

Although standard coagulation markers remain the mainstay of pathway analysis (PT, APTT), they have several limitations in assessing diseases and treatment thereof. The markers only indicate isolated parts of the plasma-based coagulation pathways. Furthermore, many of these pathway markers do not take into account the effect exerted by the cellular components in blood (such as platelets, and white and red blood cells). Furthermore, many of the tests are carried out in physiologically altered blood (in tubes with, e.g., heparin or sodium citrate). The testing process varies from laboratory to laboratory often with different reference ranges, making interpretation in various disease states difficult (Ebner et al., 2018). In addition, many of the hypercoagulable disease states have a marked inflammatory component and response due to endothelial change and damage, which can neither be measured nor assessed with these tests. In most circumstances, this makes their overall utility in many diseases mainly an adjunct to clinical care.

### Clot Microstructure

Due to the need for a global marker to assess hemostatic competency in hypercoagulable states and their treatment, recent studies have focused on the potential of clot microstructure and quality as a more global and accurate measurement. This has led to the development of a potential and exciting new marker, fractal dimension, which has been seen as an improved marker of coagulation that has the ability to assess the direct and indirect effect of coagulation in one simple and immediate test in unaltered whole blood (Kopytek et al., 2019).

**FIGURE 1:**
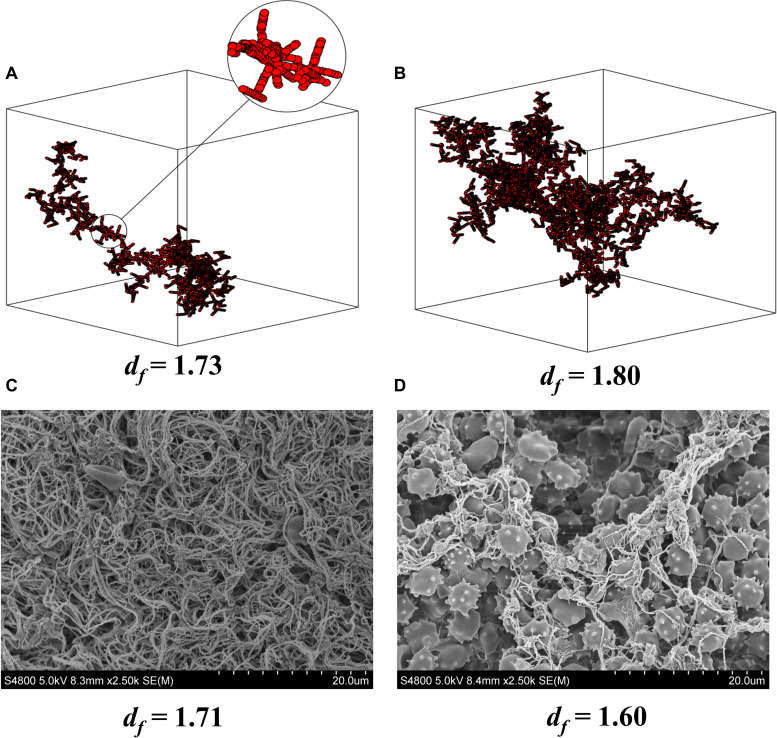
d_f_ and clot microstructure. Computer modeling of fractal structures of blood clots at the gel point from **(A)** healthy individuals and **(B)** individuals with vascular inflammatory disease. Electron microscopy images of fractal dimension (d_f_) and clot microstructure in whole blood **(C)** pre and **(D)** post 1 wk of oral dual antiplatelet therapy (75 mg Aspirin and 10 mg Prasugrel) in healthy individuals. The pictures **(C,D)** clearly show how inhibition of platelet activity alters clot microstructure and mass as indicated by d_f_. Note that a small change in d_f_ results in a large increase in mass at the gel point for the developing clot. **(A,B)** are reproduced from Curtis et al. (2011).

**FIGURE 2:**
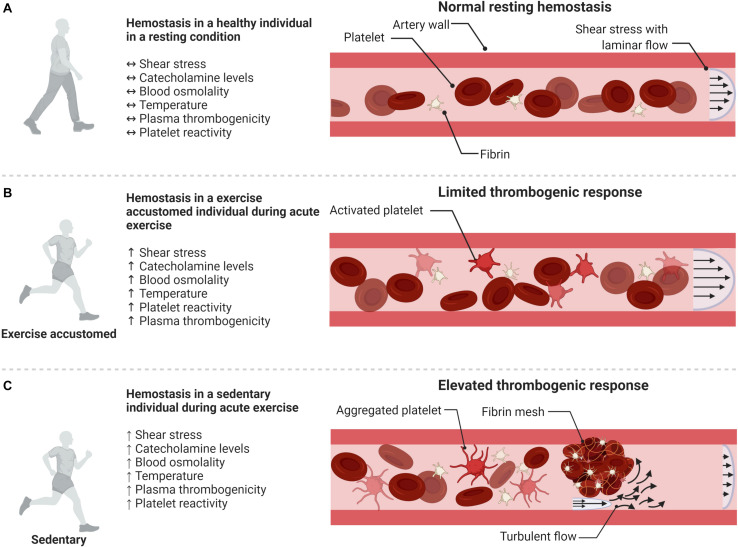
Schematic illustration of the influence of acute exercise and habitually active lifestyle on hemostasis. **(A)** Resting hemostasis in a healthy individual. **(B)** Exercise accustomed individual: exercise-induced pro-thrombotic factors are counterbalanced by the release of anti-thrombotic and anti-aggregatory agents, e.g., prostacyclin and NO. **(C)** Sedentary individual: the exercise-induced increase in thrombogenic factors is not sufficiently counterbalanced by anti-thrombotic protection leading to a thrombogenic response. Illustration created with BioRender.

Fractal dimension provides information about the structure of the fibrin network of a developing blood clot (Evans et al., 2010) and is thus a functional biomarker of hemostasis and clot microstructure. Fractal dimension provides a description of the incipient clot, which is the initial templating structure of the clot, which leads to its ongoing mechanical structure and strength (Figure 1) (Curtis et al., 2011, 2013). Low values of fractal dimension are equivalent to a weak and less dense clot with low number of branches, whereas high values are equivalent to a strong and dense clot with a high number of complex structured branches, which is more difficult to break down therapeutically (Evans et al., 2010; Sabra et al., 2017). In acute vascular inflammatory disease such as ischemic stroke and myocardial infarction, there is considerable over-production of mass and cross-linking at this templating phase, which leads to a very strong and abnormally structured clot, as compared to clot formation in healthy individuals (Figures 1, 2). In contrast to standard and conventional coagulation measurements like PT or APTT, fractal dimension is performed in untreated blood within minutes after blood withdrawal and provides a rapid measurement of the integrated hemostatic property and thereby an immediate indication of the risk of cardiovascular events (Sabra et al., 2017). Limitations include the requirement of relatively expensive equipment, and as with platelet aggregometry, the measurement is made *in vitro* without physiological hemodynamic impact. Also, the method is still relatively new, and although the method has been used in a large number of subjects (Evans et al., 2010; Lawrence et al., 2014), normal ranges for gel point and fractal dimension in different populations have yet to be defined.

## Acute Exercise and Arterial Thrombosis

Paradoxically, although regular exercise or a physically active lifestyle protect against cardiovascular disease (Albert et al., 2000; Lee et al., 2003; Sattelmair et al., 2011), a single acute bout of physical exertion might trigger an acute myocardial infarct or stroke (Lee et al., 2003). The increased risk is reported to last up to 30–60 min after termination of exercise (Röcker et al., 1986; Mittleman et al., 1993; Albert et al., 2000; Buckley et al., 2019), with one study reporting the effect to persist for up to 2 h (von Klot et al., 2008). However, an important aspect is that, whereas in a sedentary individual the risk of a myocardial infarct increases ∼ 50- to 100-fold with acute vigorous physical activity, in an individual accustomed to exercise the increased risk is much lower (∼2- to 5-fold) (Figure 2) (Bärtsch, 1999; Thompson et al., 2007). This would suggest that once an individual is accustomed to exercise, the susceptibility to thrombosis by acute exercise is substantially reduced. This aspect is further discussed below in the section on exercise training.

Exercise intensity is likely to influence the susceptibility to arterial thrombosis and may also affect the time required for returning to resting hemostasis (Supplementary Table 1) (Hegde et al., 2001; Menzel and Hilberg, 2011). As such, low to moderate intensity exercise elicits low or no increase in plasma thrombotic markers and platelet reactivity (Hegde et al., 2001; Davies et al., 2016; Lundberg Slingsby et al., 2018), whereas strenuous exercise leads to a substantial increase (Hegde et al., 2001; Davies et al., 2016). The increased risk of myocardial infarct correlates with high self-reported physical exertion, with a score representing vigorous exertion resulting in a >5-fold increase in the relative risk of onset of a myocardial infarct in the 1–2 h following exercise (Buckley et al., 2019).

One reason for the increased relative risk of arterial thrombosis with intense exercise could be elevated vascular shear stress (Badiei et al., 2015) due to the higher blood flows with intense exercise (Hathcock, 2006; Chen et al., 2010; Badiei et al., 2015). High shear stress levels can promote thrombosis as the frictional force may cause tearing or rupture of an atherosclerotic plaque (Hallqvist et al., 2000; Thompson et al., 2007). Also, many platelet activating factors are influenced by increased exercise intensities, such as catecholamines (Dimsdale and Moss, 1980; Lawrence et al., 2018), temperature (Meyer et al., 2013; Lawrence et al., 2016), blood osmolality (Lawrence et al., 2014), and central hypovolemia (Meyer et al., 2013). In addition, aspects such as endothelial alterations, which influence platelet adhesion and thrombin generation, may also be affected by exercise and lead to an increased susceptibility to thrombosis.

Catecholamines promote thrombosis by activation of platelets and an increase in clot microstructure (Mittleman et al., 1995; Thompson et al., 2007; Davies et al., 2016; Lawrence et al., 2018). In a recent study, tyramine infusion, leading to endogenous noradrenalin formation, was found to increase fractal dimension, indicating a denser incipient clot microstructure (Lawrence et al., 2018). Accordingly, compared to moderate intensity exercise, intense exercise with higher plasma norepinephrine concentrations was shown to be associated with significantly higher levels of thrombotic plasma markers (Menzel and Hilberg, 2011). Nevertheless, single leg knee extensor exercise, which has a limited impact on sympathetic activity, significantly increases fractal dimension suggesting that local hemodynamic changes such as shear stress can influence clot microstructure independently of catecholamines (Lawrence et al., 2018). Combined these studies provide support for that both catecholamines and hemodynamic changes during exercise can influence the microstructure of developing blood clots, but further studies elucidating their precise role are required.

Data on exercise-induced effects in healthy individuals may differ from data in patient groups for whom the exercise-induced thrombogenicity is likely to be more critical. For example, although patients with either hypertension or coronary artery disease do not seem to differ in basal platelet reactivity or markers of thrombosis compared to healthy controls (Petidis et al., 2008; Hong et al., 2009), these patient groups may present increased catecholamine levels during and after exercise (Petidis et al., 2008) with a potential impact on thrombogenicity. Nevertheless, findings on exercise-induced thrombogenicity in coronary artery disease patients are inconsistent (Mehta and Mehta, 1982; Pamukcu et al., 2005; Aurigemma et al., 2007; Petidis et al., 2008), likely due to variations in criteria for medication and co-morbidities of the populations.

It should be emphasized that despite the apparent immediate changes in thrombogenicity after exercise, the risk of hospitalization during clinical graded exercise testing is approximately one in 10.000 (Myers et al., 2009). In healthy adults, between 30 and 60 years of age, this risk of a cardiac event is estimated to be ∼0.2 and 0.3 per 10.000 person-hours of exercise for women and men, respectively (Gibbons et al., 1980). Moreover, in one of the few studies assessing platelet reactivity following acute low intensity one-leg knee extensor exercise, a significant decrease in epinephrine-induced platelet aggregation was observed in moderately and highly trained, but not sedentary, middle-aged male subjects (Lundberg Slingsby et al., 2018). This finding suggests that, in individuals accustomed to exercise, the risk of arterial thrombosis after exercise of lower intensities may even be lower than at rest. Also, plasma fibrinolysis has been shown to increase after acute exercise as a result of increased release of t-PA from endothelial cells in the vasculature (Rankinen et al., 1995; Gunga et al., 2002; Sumann et al., 2007). The increase in t-PA occurs with different exercise modalities but appears to be higher with higher exercise intensity (Handa et al., 1992; El-Sayed, 1993; Hegde et al., 2001; Menzel and Hilberg, 2011).

## Influence of Exercise Training on Susceptibility to Arterial Thrombosis

It is well established that regular physical activity protects against cardiovascular disease (Buckley et al., 2019), and physical activity for ≥ 30 minutes per day, 5 days a week, is advised (Arnett et al., 2019; Pelliccia et al., 2020). One of the beneficial effects of regular physical activity on arterial thrombi formation is likely through a reduction in basal platelet reactivity (Lundberg Slingsby et al., 2018; Heber et al., 2020). Whereas untrained individuals display an increased level of adrenaline-induced platelet aggregation in response to an acute bout of exercise, platelet aggregation remains unaffected in moderately trained or well-trained individuals (Lundberg Slingsby et al., 2018). Similar to that in healthy individuals, exercise training in patients with coronary artery disease has been found to decrease platelet reactivity, and inclusion of high intensity exercise sessions has been reported to be more beneficial than moderate exercise training alone (Heber et al., 2020). This effect of reduced platelet reactivity with the added high intensity exercise sessions is also present immediately after acute exercise (Supplementary Table 3) (Heber et al., 2020).

Currently, only a limited amount of data exists regarding the influence of exercise training on the thrombogenic clotting profile (Supplementary Tables 2, 3) (Stratton et al., 1991; van den Burg et al., 2000; Lundberg Slingsby et al., 2017, 2018; Heber et al., 2020). In these studies, exercise training consistently resulted in reduced platelet reactivity and a reduced level of plasma markers indicating thrombogenicity (Rauramaa et al., 1986; El-Sayed et al., 1995; Wang et al., 1995, 1997; van den Burg et al., 2000; Lundberg Slingsby et al., 2017; Heber et al., 2020). Interestingly, in late premenopausal women, a period of aerobic interval training by cycling, reduced platelet reactivity, whereas this effect was not observed in recently postmenopausal women (Lundberg Slingsby et al., 2017). The reason for this discrepancy is unclear. It should be noted that both groups of women were found to have improved endothelial function as well as enhanced sensitivity to platelet inhibition by prostacyclin. Improved endothelial function is associated with enhanced formation of prostacyclin and nitric oxide (Taddei et al., 2006; Flammer et al., 2012) which both are potent inhibitors of platelet reactivity (Jin et al., 2005; Yau et al., 2015). Several studies, including studies on pre- and postmenopausal women, have shown that exercise training leads to increased expression of enzymes related to prostacyclin synthesis and nitric oxide formation as well as increased plasma levels of prostacyclin and nitric oxide metabolites (Hellsten et al., 2012; Cocks et al., 2013; Nyberg et al., 2017).

Thus, although numerous studies have examined the effect of regular physical activity on cardiovascular risk factors, the specific influence of physical activity on thrombogenicity has not been well investigated, and in particular, the role of exercise intensity and volume is lacking. Future studies should therefore aim to assess the impact of differentiated training modalities to evaluate the influence on plasma markers of hemostasis, platelet aggregation, and clot microstructure.

## Conclusion

Exercise may be considered a double-edged sword since, on one hand, acute exercise can be a direct cause of a thrombotic event, and on the other hand, exercise training is a potent intervention for lowering the risk of cardiovascular events. Although further studies are required to unravel the influence of different exercise modalities, existing literature points at a greater risk of enhanced thrombogenicity with high, rather than light to moderate intensity exercise. Thus, for patients at risk, the safer recommendation would accordingly be to initiate exercise programs at low to moderate intensities. We propose that studies combining platelet reactivity assay, clinical measures of hemostatic markers, and the novel functional measure of clot microstructure will provide a new level of detailed prediction of the susceptibility to harmful arterial blood clots.

## Author Contributions

LO and MF drafted the manuscript. LG, PE, and YH critically revised and contributed to the content of the manuscript and approved its final version. All authors contributed to the article and approved the submitted version.

## Conflict of Interest

The authors declare that the research was conducted in the absence of any commercial or financial relationships that could be construed as a potential conflict of interest.

## Supplementary Material

Supplementary Table 1 Influence of an acute exercise bout on biomarkers of thrombogenicity.

Supplementary Table 2 The effect of different training protocols on biomarkers of thrombogenicity.

Supplementary Table 3 Influence of an acute exercise bout on biomarkers of thrombogenicity in patient groups compared to healthy controls.

## References

[B1] Albert C. M. Mittleman M. A. Chae C. U. Lee I. M. Hennekens C. H. Manson J. E. (2000). Triggering of sudden death from cardiac causes by vigorous exertion. *N. Engl. J. Med.* 343 1355–1361. 10.1056/NEJM200011093431902 11070099

[B2] Algahtani M. Heptinstall S. (2017). Novel strategies for assessing platelet reactivity. *Future Cardiol.* 13 33–47. 10.2217/fca-2016-0054 27990840

[B3] Arnett D. K. Blumenthal R. S. Albert M. A. Buroker A. B. Goldberger Z. D. Hahn E. J. (2019). 2019 ACC/AHA guideline on the primary prevention of cardiovascular disease: a report of the American College of Cardiology/American Heart Association task force on clinical practice guidelines. *Circulation* 140 e596–e646. 10.1161/CIR.0000000000000678 30879355 PMC7734661

[B4] Aurigemma C. Fattorossi A. Sestito A. Sgueglia G. A. Farnetti S. Buzzonetti A. (2007). Relationship between changes in platelet reactivity and changes in platelet receptor expression induced by physical exercise. *Thromb. Res.* 120 901–909. 10.1016/j.thromres.2007.01.009 17337041

[B5] Badiei N. Sowedan A. M. Curtis D. J. Brown M. R. Lawrence M. J. Campbell A. I. (2015). Effects of unidirectional flow shear stresses on the formation, fractal microstructure and rigidity of incipient whole blood clots and fibrin gels. *Clin. Hemorheol. Microcirc.* 60 451–464. 10.3233/CH-151924 25624413 PMC4923731

[B6] Bärtsch P. (1999). Platelet activation with exercise and risk of cardiac events. *Lancet* 354 1747–1748. 10.1016/S0140-6736(99)90259-3 10577632

[B7] Born G. V. (1962). Aggregation of blood platelets by adenosine diphosphate and its reversal. *Nature* 194 927–929. 10.1038/194927b0 13871375

[B8] Buckley T. Soo Hoo S. Y. Shaw E. Hansen P. S. Fethney J. Tofler G. H. (2019). Triggering of acute coronary occlusion by episodes of vigorous physical exertion. *Heart Lung Circ.* 28 1773–1779. 10.1016/j.hlc.2018.11.001 30555009

[B9] Chen Y. W. Chen J. K. Wang J. S. (2010). Strenuous exercise promotes shear-induced thrombin generation by increasing the shedding of procoagulant microparticles from platelets. *Thromb. Haemost.* 104 293–301. 10.1160/TH09-09-0633 20589321

[B10] Cocks M. Shaw C. S. Shepherd S. O. Fisher J. P. Ranasinghe A. M. Barker T. A. (2013). Sprint interval and endurance training are equally effective in increasing muscle microvascular density and eNOS content in sedentary males. *J. Physiol.* 591 641–656. 10.1113/jphysiol.2012.239566 22946099 PMC3577551

[B11] Collet J. P. Allali Y. Lesty C. Tanguy M. L. Silvain J. Ankri A. (2006). Altered fibrin architecture is associated with hypofibrinolysis and premature coronary atherothrombosis. *Arterioscler. Thromb. Vasc. Biol.* 26 2567–2573. 10.1161/01.ATV.0000241589.52950.4c 16917107

[B12] Collet J. P. Park D. Lesty C. Soria J. Soria C. Montalescot G. (2000). Influence of fibrin network conformation and fibrin fiber diameter on fibrinolysis speed: dynamic and structural approaches by confocal microscopy. *Arterioscler. Thromb. Vasc. Biol.* 20 1354–1361. 10.1161/01.ATV.20.5.1354 10807754

[B13] Collins P. Ford I. Ball D. Macaulay E. Greaves M. Brittenden J. (2006). A preliminary study on the effects of exercising to maximum walking distance on platelet and endothelial function in patients with intermittent claudication. *Eur. J. Vasc. Endovasc. Surg.* 31 266–273. 10.1016/j.ejvs.2005.10.011 16360327

[B14] Curtis D. J. Brown M. R. Hawkins K. Evans P. A. Lawrence M. J. Rees P. (2011). Rheometrical and molecular dynamics simulation studies of incipient clot formation in fibrin-thrombin gels: an activation limited aggregation approach. *J. Nonnewton. Fluid Mech.* 166 932–938. 10.1016/j.jnnfm.2011.04.016

[B15] Curtis D. J. Williams P. R. Badiei N. Campbell A. I. Hawkins K. Evans P. A. (2013). A study of microstructural templating in fibrin–thrombin gel networks by spectral and viscoelastic analysis. *Soft Matter* 9 4883–4889. 10.1039/c3sm50263e

[B16] Davidson J. F. Colvin B. T. Barrowcliffe T. W. Dawson D. W. Machin S. J. Poller L. (1988). Guidelines on platelet function testing. The British society for haematology BCSH haemostasis and thrombosis task force. *J. Clin. Pathol.* 41 1322–1330. 10.1136/jcp.41.12.1322 3225335 PMC1141768

[B17] Davies N. A. Llwyd O. Brugniaux J. V. Davies G. R. Marley C. J. Hodson D. (2016). Effects of exercise intensity on clot microstructure and mechanical properties in healthy individuals. *Thromb. Res.* 143 130–136. 10.1016/j.thromres.2016.1005.1018 27240111

[B18] de Groot P. G. Urbanus R. T. Roest M. (2012). Platelet interaction with the vessel wall. *Handb. Exp. Pharmacol.* 210 87–110. 10.1007/978-3-642-29423-5_4 22918728

[B19] Dimsdale J. E. Moss J. (1980). Plasma catecholamines in stress and exercise. *JAMA* 243 340–342. 10.1001/jama.1980.03300300018017 7351746

[B20] Ebner M. Birschmann I. Peter A. Härtig F. Spencer C. Kuhn J. (2018). Limitations of specific coagulation tests for direct oral anticoagulants: a critical analysis. *J. Am. Heart Assoc.* 7:e009807. 10.1161/JAHA.118.009807 30371316 PMC6404908

[B21] El-Sayed M. S. (1993). Fibrinolytic and hemostatic parameter response after resistance exercise. *Med. Sci. Sports Exerc.* 25 597–602. 10.1249/00005768-199305000-00011 8492688

[B22] El-Sayed M. S. Lin X. Rattu A. J. (1995). Blood coagulation and fibrinolysis at rest and in response to maximal exercise before and after a physical conditioning programme. *Blood Coagul. Fibrinolysis* 6 747–752. 10.1097/00001721-199512000-00009 8825226

[B23] Ersöz G. Zergeroğlu A. M. Yakaryilmaz A. (2002). The effect of submaximal exercise on platelet aggregation during late follicular and midluteal phases in women. *Thromb. Res.* 108 147–150. 10.1016/S0049-3848(02)00404-8 12590951

[B24] Evans P. A. Hawkins K. Morris R. H. Thirumalai N. Munro R. Wakeman L. (2010). Gel point and fractal microstructure of incipient blood clots are significant new markers of hemostasis for healthy and anticoagulated blood. *Blood* 116 3341–3346. 10.1182/blood-2010-02-269324 20566899

[B25] Feng L. Zhao Y. Zhao H. Shao Z. (2014). Effects of storage time and temperature on coagulation tests and factors in fresh plasma. *Sci. Rep.* 4:3868. 10.1038/srep03868 24463857 PMC3902390

[B26] Flammer A. J. Anderson T. Celermajer D. S. Creager M. A. Deanfield J. Ganz P. (2012). The assessment of endothelial function: from research into clinical practice. *Circulation* 126 753–767. 10.1161/CIRCULATIONAHA.112.093245 22869857 PMC3427943

[B27] Gibbons L. W. Cooper K. H. Meyer B. M. Ellison R. C. (1980). The acute cardiac risk of strenuous exercise. *JAMA* 244 1799–1801. 10.1001/jama.1980.03310160015015 7420679

[B28] Gunga H. C. Kirsch K. Beneke R. Böning D. Hopfenmüller W. Leithäuser R. (2002). Markers of coagulation, fibrinolysis and angiogenesis after strenuous short-term exercise (Wingate-test) in male subjects of varying fitness levels. *Int. J. Sports Med.* 23 495–499. 10.1055/s-2002-35070 12402181

[B29] Hallqvist J. Möller J. Ahlbom A. Diderichsen F. Reuterwall C. De Faire U. (2000). Does heavy physical exertion trigger myocardial infarction? A case-crossover analysis nested in a population-based case-referent study. *Am. J. Epidemiol.* 151 459–467. 10.1093/oxfordjournals.aje.a010231 10707914

[B30] Handa K. Terao Y. Mori T. Tanaka H. Kiyonaga A. Matsunaga A. (1992). Different coagulability and fibrinolytic activity during exercise depending on exercise intensities. *Thromb. Res.* 66 613–616. 10.1016/0049-3848(92)90317-4 1523616

[B31] Hathcock J. J. (2006). Flow effects on coagulation and thrombosis. *Arterioscler. Thromb. Vasc. Biol.* 26 1729–1737. 10.1161/01.ATV.0000229658.76797.30 16741150

[B32] Heber S. Fischer B. Sallaberger-Lehner M. Hausharter M. Ocenasek H. Gleiss A. (2020). Effects of high-intensity interval training on platelet function in cardiac rehabilitation: a randomised controlled trial. *Heart* 106 69–79. 10.1136/heartjnl-2019-315130 31315940

[B33] Hegde S. S. Goldfarb A. H. Hegde S. (2001). Clotting and fibrinolytic activity change during the 1 h after a submaximal run. *Med. Sci. Sports Exerc.* 33 887–892. 10.1097/00005768-200106000-00006 11404652

[B34] Hellsten Y. Jensen L. Thaning P. Nyberg M. Mortensen S. (2012). Impaired formation of vasodilators in peripheral tissue in essential hypertension is normalized by exercise training: role of adenosine and prostacyclin. *J. Hypertens* 30 2007–2014. 10.1097/HJH.0b013e328356dd57 22902871

[B35] Hong S. Adler K. A. Von Känel R. Nordberg J. Ziegler M. G. Mills P. J. (2009). Prolonged platelet activation in individuals with elevated blood pressure in response to a moderate exercise challenge. *Psychophysiology* 46 276–284. 10.1111/j.1469-8986.2008.00779.x 19170949 PMC2724895

[B36] Jin R. C. Voetsch B. Loscalzo J. (2005). Endogenous mechanisms of inhibition of platelet function. *Microcirculation* 12 247–258. 10.1080/10739680590925493 15814434

[B37] Koenig W. Ernst E. (2000). Exercise and thrombosis. *Coron. Artery. Dis.* 11 123–127. 10.1097/00019501-200003000-00006 10758813

[B38] Kopytek M. Zabczyk M. Natorska J. Siudut J. Malinowski K. P. Ptaszek P. (2019). Viscoelastic properties of plasma fibrin clots are similar in patients on rivaroxaban and vitamin K antagonists. *J. Physiol. Pharmacol.* 70 79–85. 10.26402/jpp.2019.1.05 31019123

[B39] Lawrence M. J. Davies G. Nyberg M. Whitley J. Evans V. Williams R. (2018). The effect of tyramine infusion and exercise on blood flow, coagulation and clot microstructure in healthy individuals. *Thromb. Res.* 170 32–37. 10.1016/j.thromres.2018.1007.1025 30098458

[B40] Lawrence M. J. Kumar S. Hawkins K. Boden S. Rutt H. Mills G. (2014). A new structural biomarker that quantifies and predicts changes in clot strength and quality in a model of progressive haemodilution. *Thromb. Res.* 134 488–494. 10.1016/j.thromres.2014.05.039 24965661

[B41] Lawrence M. J. Marsden N. Mothukuri R. Morris R. H. Davies G. Hawkins K. (2016). The effects of temperature on clot microstructure and strength in healthy volunteers. *Anesth. Analg.* 122 21–26. 10.1213/ANE.0000000000000992 26440418

[B42] Lee C. D. Folsom A. R. Blair S. N. (2003). Physical activity and stroke risk: a meta-analysis. *Stroke* 34 2475–2481. 10.1161/01.STR.0000091843.02517.9D 14500932

[B43] Lundberg Slingsby M. H. Gliemann L. Thrane M. Rytter N. Egelund J. Chan M. V. (2018). Platelet responses to pharmacological and physiological interventions in middle-aged men with different habitual physical activity levels. *Acta Physiol. (Oxf.)* 223:e13028. 10.1111/apha.13028 29297976

[B44] Lundberg Slingsby M. H. Nyberg M. Egelund J. Mandrup C. M. Frikke-Schmidt R. Kirkby N. S. (2017). Aerobic exercise training lowers platelet reactivity and improves platelet sensitivity to prostacyclin in pre- and postmenopausal women. *J. Thromb. Haemost.* 15 2419–2431. 10.1111/jth.13866 29027349

[B45] Mackman N. (2008). Triggers, targets and treatments for thrombosis. *Nature* 451 914–918. 10.1038/nature06797 18288180 PMC2848509

[B46] Mehta J. Mehta P. (1982). Comparison of platelet function during exercise in normal subjects and coronary artery disease patients:potential role of platelet activation in myocardial ischemia. *Am. Heart J.* 103 49–53. 10.1016/0002-8703(82)90528-2 6172974

[B47] Menzel K. Hilberg T. (2011). Blood coagulation and fibrinolysis in healthy, untrained subjects: effects of different exercise intensities controlled by individual anaerobic threshold. *Eur. J. Appl. Physiol.* 111 253–260. 10.1007/s00421-010-1640-2 20859637

[B48] Meyer M. A. Ostrowski S. R. Overgaard A. Ganio M. S. Secher N. H. Crandall C. G. (2013). Hypercoagulability in response to elevated body temperature and central hypovolemia. *J. Surg. Res.* 185 e93–e100. 10.1016/j.jss.2013.06.012 23856126 PMC4961036

[B49] Mills J. D. Ariëns R. A. Mansfield M. W. Grant P. J. (2002). Altered fibrin clot structure in the healthy relatives of patients with premature coronary artery disease. *Circulation* 106 1938–1942. 10.1161/01.CIR.0000033221.73082.06 12370216

[B50] Mittleman M. A. Maclure M. Sherwood J. B. Mulry R. P. Tofler G. H. Jacobs S. C. (1995). Triggering of acute myocardial infarction onset by episodes of anger. Determinants of myocardial infarction onset study investigators. *Circulation* 92 1720–1725. 10.1161/01.CIR.92.7.1720 7671353

[B51] Mittleman M. A. Maclure M. Tofler G. H. Sherwood J. B. Goldberg R. J. Muller J. E. (1993). Triggering of acute myocardial infarction by heavy physical exertion. Protection against triggering by regular exertion. Determinants of myocardial infarction onset study investigators. *N. Engl. J. Med.* 329 1677–1683. 10.1056/NEJM199312023292301 8232456

[B52] Mozaffarian D. Benjamin E. J. Go A. S. Arnett D. K. Blaha M. J. Cushman M. (2016). Executive summary: heart disease and stroke statistics–2016 update: a report from the American Heart Association. *Circulation* 133 447–454. 10.1161/CIR.0000000000000366 26811276

[B53] Myers J. Arena R. Franklin B. Pina I. Kraus W. E. Mcinnis K. (2009). Recommendations for clinical exercise laboratories: a scientific statement from the american heart association. *Circulation* 119 3144–3161. 10.1161/CIRCULATIONAHA.109.192520 19487589

[B54] Nachman R. L. Rafii S. (2008). Platelets, petechiae, and preservation of the vascular wall. *N. Engl. J. Med.* 359 1261–1270. 10.1056/NEJMra0800887 18799560 PMC2935201

[B55] Nyberg M. Egelund J. Mandrup C. M. Andersen C. B. Hansen K. Hergel I. F. (2017). Leg vascular and skeletal muscle mitochondrial adaptations to aerobic high-intensity exercise training are enhanced in the early postmenopausal phase. *J. Physiol.* 595 2969–2983. 10.1113/JP273871 28231611 PMC5407965

[B56] O’brien J. R. (1962). Platelet aggregation: part I some effects of the adenosine phosphates, thrombin, and cocaine upon platelet adhesiveness. *J. Clin. Pathol.* 15 446–452. 10.1136/jcp.15.5.446 16810985 PMC480432

[B57] Pamukcu B. Oflaz H. Acar R. D. Umman S. Koylan N. Umman B. (2005). The role of exercise on platelet aggregation in patients with stable coronary artery disease: exercise induces aspirin resistant platelet activation. *J. Thromb. Thrombolysis* 20 17–22. 10.1007/s11239-005-2318-1 16133890

[B58] Paniccia R. Priora R. Liotta A. A. Abbate R. (2015). Platelet function tests: a comparative review. *Vasc. Health Risk Manag.* 11 133–148. 10.2147/VHRM.S44469 25733843 PMC4340464

[B59] Pelliccia A. Sharma S. Gati S. Bäck M. Börjesson M. Caselli S. (2020). 2020 ESC guidelines on sports cardiology and exercise in patients with cardiovascular disease: the task force on sports cardiology and exercise in patients with cardiovascular disease of the European Society of Cardiology (ESC). *Eur. Heart J.* 42 17–96.

[B60] Petidis K. Douma S. Doumas M. Basagiannis I. Vogiatzis K. Zamboulis C. (2008). The interaction of vasoactive substances during exercise modulates platelet aggregation in hypertension and coronary artery disease. *BMC Cardiovasc. Disord* 8:11. 10.1186/1471-2261-8-11 18505546 PMC2432046

[B61] Rankinen T. Väisänen S. Penttilä I. Rauramaa R. (1995). Acute dynamic exercise increases fibrinolytic activity. *Thromb. Haemost.* 73 281–286. 10.1055/s-0038-1653765 7792744

[B62] Rauramaa R. Salonen J. T. Seppänen K. Salonen R. Venäläinen J. M. Ihanainen M. (1986). Inhibition of platelet aggregability by moderate-intensity physical exercise: a randomized clinical trial in overweight men. *Circulation* 74 939–944. 10.1161/01.CIR.74.5.939 3533315

[B63] Röcker L. Drygas W. K. Heyduck B. (1986). Blood platelet activation and increase in thrombin activity following a marathon race. *Eur. J. Appl. Physiol. Occup. Physiol.* 55 374–380. 10.1007/BF00422736 3758037

[B64] Sabra A. Lawrence M. J. Aubrey R. Obaid D. Chase A. Smith D. (2017). Characterisation of clot microstructure properties in stable coronary artery disease. *Open Heart* 4:e000562. 10.1136/openhrt-2016-000562 28761676 PMC5515126

[B65] Salehi S. H. Fatemi M. J. Asadi K. Shoar S. Ghazarian A. D. Samimi R. (2014). Electrical injury in construction workers: a special focus on injury with electrical power. *Burns* 40 300–304. 10.1016/j.burns.2013.05.019 23816398

[B66] Sattelmair J. Pertman J. Ding E. L. Kohl H. W. III Haskell W. Lee I. M. (2011). Dose response between physical activity and risk of coronary heart disease: a meta-analysis. *Circulation* 124 789–795. 10.1161/CIRCULATIONAHA.110.010710 21810663 PMC3158733

[B67] Stratton J. R. Chandler W. L. Schwartz R. S. Cerqueira M. D. Levy W. C. Kahn S. E. (1991). Effects of physical conditioning on fibrinolytic variables and fibrinogen in young and old healthy adults. *Circulation* 83 1692–1697. 10.1161/01.CIR.83.5.1692 1902407

[B68] Sumann G. Fries D. Griesmacher A. Falkensammer G. Klingler A. Koller A. (2007). Blood coagulation activation and fibrinolysis during a downhill marathon run. *Blood Coagul. Fibrinolysis* 18 435–440. 10.1097/MBC.0b013e328136c19b 17581317

[B69] Taddei S. Virdis A. Ghiadoni L. Versari D. Salvetti A. (2006). Endothelium, aging, and hypertension. *Curr. Hypertens. Rep.* 8 84–89. 10.1007/s11906-006-0045-4 16600164

[B70] Thompson P. D. Franklin B. A. Balady G. J. Blair S. N. Corrado D. Estes N. A. III (2007). Exercise and acute cardiovascular events placing the risks into perspective: a scientific statement from the American Heart Association Council on Nutrition, physical activity, and metabolism and the council on clinical cardiology. *Circulation.* 115 2358–2368. 10.1161/CIRCULATIONAHA.107.181485 17468391

[B71] Thompson W. R. Sallis R. Joy E. Jaworski C. A. Stuhr R. M. Trilk J. L. (2020). Exercise Is Medicine. *Am. J. Lifestyle Med.* 14 511–523. 10.1177/1559827620912192 32922236 PMC7444006

[B72] van den Burg P. J. Hospers J. E. Mosterd W. L. Bouma B. N. Huisveld I. A. (2000). Aging, physical conditioning, and exercise-induced changes in hemostatic factors and reaction products. *J. Appl. Physiol. (1985)* 88 1558–1564. 10.1152/jappl.2000.88.5.1558 10797112

[B73] Vinholt P. J. Hvas A. M. Nybo M. (2014). An overview of platelet indices and methods for evaluating platelet function in thrombocytopenic patients. *Eur. J. Haematol.* 92 367–376. 10.1111/ejh.12262 24400878

[B74] Vinholt P. J. Nybo M. Nielsen C. B. Hvas A. M. (2017). Light transmission aggregometry using pre-coated microtiter plates and a Victor X5 plate reader. *PLoS One* 12:e0185675. 10.1371/journal.pone.0185675 29023589 PMC5638243

[B75] von Klot S. Mittleman M. A. Dockery D. W. Heier M. Meisinger C. Hörmann A. (2008). Intensity of physical exertion and triggering of myocardial infarction: a case-crossover study. *Eur. Heart J.* 29 1881–1888. 10.1093/eurheartj/ehn235 18534976

[B76] Wang J. S. Jen C. J. Chen H. I. (1995). Effects of exercise training and deconditioning on platelet function in men. *Arterioscler. Thromb. Vasc. Biol.* 15 1668–1674. 10.1161/01.ATV.15.10.1668 7583542

[B77] Wang J. S. Jen C. J. Chen H. I. (1997). Effects of chronic exercise and deconditioning on platelet function in women. *J. Appl. Physiol. (1985)* 83 2080–2085. 10.1152/jappl.1997.83.6.2080 9390984

[B78] Yau J. W. Teoh H. Verma S. (2015). Endothelial cell control of thrombosis. *BMC Cardiovasc Disord.* 15:130. 10.1186/s12872-015-0124-z 26481314 PMC4617895

