# Supplementary material for: Does Exercise Influence the Susceptibility to Arterial Thrombosis? An Integrative Perspective
Source: Front Physiol. 2021 Feb 23;12:636027. doi: 10.3389/fphys.2021.636027 (PMC7940832; doi:10.3389/fphys.2021.636027)
Supplement: Supplementary Table 1 — Influence of an acute exercise bout on biomarkers of thrombogenicity. [file Table_1.docx]

Table. 1. Influence of an acute exercise bout on biomarkers of thrombogenicity.

| **Subject characteristics** | **Exercise mode // duration // intensity** | **Biochemical markers of blood homeostasis: Post** **vs. pre acute** **exercise** | **Reference** |
| --- | --- | --- | --- |
| **Males and Females** **(grouped)**  21 Males & 3 Females  Age: 26 ± 7 yr.  VO2_max_ (ml kg min^-1^): 35.6 ± 7.3 | Semi-recumbent bike ergometer // 5 min (submaximal) & ~12 min (maximal) //  Submaximal Exercise (5 watts)  Maximal Exercise (peak power 269 ± 48 watts) | (arrows indicate **vs. pre** resting plasma markers)   \| **Submaximal** Exercise  ↔ d_f_  ↔ T_GP_  ↔ G_GP_  ↔ PT  ↔ APTT  ↔ Clauss  ↔ Fibrinogen  ↔ D-dimer  ↔ Factor VIII:C  ↔ vWF Antigen  ↔ PAI-1 Antigen \| **Maximal** Exercise  ↑ d_f_  ↓ T_GP_  ↑ G_GP_  ↔ PT  ↓ APTT  ↔ Clauss  ↔ Fibrinogen  ↔ D-dimer  ↔ Factor VIII:C  ↔ vWF Antigen  ↑ PAI-1 Antigen \| \| --- \| --- \| | (Davies et al., 2016) (n=24) |
| **Male only**  Age: 25 ± 3 yr.  VO2_peak_ (ml kg min^-1^): 43.1 ± 5.2 | Bike-ergometer // 60 min // Moderate Exercise (57 ± 9 % VO2_Peak_)    Strenuous Exercise (69 ± 10 % VO2_Peak_) | (arrows indicate **vs. pre** resting plasma markers; double arrow **vs. moderately** exercise in addition)   \| **Moderate** Exercise:  ↑ D-dimer  ↑ Factor VIII  ↑ Plasmin–a2–antiplasmin complex  ↑ t-PA Activator  ↑ t-PA Antigen  ↓ APTT  ↔ PT  ↔ Post exercise Lactate  ↑ Epinephrine  ↑ Norepinephrine \| **Strenuous** Exercise:  ↓ D-dimer  ↑↑ Factor VIII  ↑↑ Plasmin–a2–antiplasmin complex  ↑↑ t-PA Activator  ↑↑ t-PA Antigen  ↓ APTT  ↔ PT  ↑ Post exercise Lactate  ↑ Epinephrine  ↑↑ Norepinephrine \| \| --- \| --- \| | (Menzel and Hilberg, 2011)  (n=20) |
| **Males Only**  Age: 23.4 ± 2.8 yr.  VO2_max_ (ml kg min^-1^): 47.8 ± 5.4 | Walk // 30 min 1.93 km h^-1^  Run // 30 min 70–75% VO2_max_ (Moderate intensity) | (arrows indicate **vs. pre** resting plasma markers)   \| **Walk**:  ↔ APTT  ↔ D-dimer  ↔ Factor VIII  ↔ t-PA \| **Run:**  ↓ APTT  ↑ D-dimer  ↑ Factor VIII  ↑ t-PA \| \| --- \| --- \| | (Hegde et al., 2001)  (n=10) |
| **Males Only**  Age: 22.9 ± 3.4 yr.  VO2_max_ (ml kg min^-1^): 41.4 ± 3.9  Blood taken immediately before ceasing of exercise. | Bike-ergometer // 20 min //  ½ of lactate threshold, lactate threshold and 4 mmol/L. | (arrows indicate **vs. pre** resting plasma markers)   \| **½ lactate threshold:**  ↔ t-PA Antigen  ↔ t-PA Activator  ↔ PT  ↔ APTT  ↔ PAI-1 Antigen  ↔ Plasminogen \| **lactate threshold:**  ↔ t-PA Antigen  ↔ t-PA Activator  ↔ PT  ↓ APTT  ↔ PAI-1 Antigen  ↔ Plasminogen \| **4 mmol/L:**  ↑ t-PA Antigen  ↑ t-PA Activator  ↔ PT  ↓ APTT  ↔ PAI-1 Antigen  ↑ Plasminogen \| \| --- \| --- \| --- \| | (Handa et al., 1992)  (n=10) |
| **Males only**  Age: 28.0 yr. (P_25_ 23/P_75_ 32)  VO2_peak_ (ml kg min^-1^): 47.62 ± (P_25_ 45,21/P_75_ 52,27)  (medians and P25/75 – percentiles) | Bike ergometer // Sprints 30sec // Wingate-testing (Maximal intensity) | (arrows indicate **vs. pre** resting plasma markers; double arrow **vs. 30 min post** in addition)   \| **2 min post**:  ↑↑ D-dimer  ↑↑ Factor VIII  ↑↑ Fibrinogen  ↑↑ PT  ↑↑ t-PA  ↓ PTT \| **9 min post**:  ↑ D-dimer  ↑↑ Factor VIII  ↑↑ Fibrinogen  ↑↑ PT  ↑↑ t-PA  ↓ PTT \| **30 min post:**  ↑ D-dimer  ↑ Factor VIII  ↑ Fibrinogen  ↑ PT  ↑ t-PA  ↓ PTT \| \| --- \| --- \| --- \| | (Gunga et al., 2002)  (n=15) |
| **Males only**  Age: 31.8 yr.  “Well-trained healthy male” | Marathon running finnish // 2:44:30 ± NA | (arrows indicate **vs. pre** resting plasma markers; double arrow **vs. 22 hours post** exercise in addition)   \| **Immediately post**:  ↑↑ Factor VIII  ↔ Fibrinogen  ↔ PT  ↓ PTT \| **60 min post**:  ↑↑ Factor VIII  ↔ Fibrinogen  ↔ PT  ↓ PTT \| **22 hours post:**  ↑ Factor VIII  ↔ Fibrinogen  ↔ PT  ↔ PTT \| \| --- \| --- \| --- \| | (Röcker et al., 1986)  (n=16) |
| **Males only**  Untrained (n=13)  Age: 52.5 ± 5.4 yr.  VO2_max_ (ml kg min^-1^): 34.5 ± 4.3  Moderately trained (n=15)  Age: 52.0 ± 5.4 yr.  VO2_max_ (ml kg min^-1^): 46.6 ± 3.9  Highly trained (n=14)  Age: 50.1 ± 5.6 yr.  VO2_max_ (ml kg min^-1^): 60.0 ± 6.0 | Dynamic one-leg knee-extension // 10 min & 10 min // 40% watt max:  17 ± 7.2 Untrained  23 ± 7.7 moderately trained  32 ± 3.7 highly trained | **Resting:** (one arrow indicates **vs. untrained** resting platelet reactivity; double arrow **vs. moderately** trained in addition)   \|  \| **Moderately trained:**  **platelet reactivity induced by:**  ↔ Collagen  ↔ Adrenaline \| **Highly trained:**  **platelet reactivity induced by:**  ↓↓ Collagen  ↓↓ Adrenaline \| \| --- \| --- \| --- \|   **12 Watt:** (arrows indicate **vs. inherent** resting platelet reactivity)   \| **Sedentary:**  ↔ Adrenaline induced platelet aggregation \| **Moderately trained:**  ↓ Adrenaline induced platelet aggregation \| **Highly trained:**  ↓ Adrenaline induced platelet aggregation \| \| --- \| --- \| --- \|   **40% Watt_max_:** (arrows indicate **vs. inherent** resting reactivity)   \| **Sedentary:**  ↑ Adrenaline induced platelet aggregation \| **Moderately trained:**  ↔ Adrenaline induced platelet aggregation \| **Highly trained:**  ↔ Adrenaline induced platelet aggregation \| \| --- \| --- \| --- \| | (Lundberg Slingsby et al., 2018)  (n=42) |
| **Female only**  14 completed  Age: 20.1 ± 1.4 yr.  VO2_max_ (ml kg min^-1^): 30.4 ± 3.2  Menstrual phase confirmed by plasma progesterone levels:   - **Late follicular** - **Midluteal** | Bike-ergometer // 15 min // Moderate intensity (75% HR_max_) | **Pre-exercise:** (arrows indicate **vs. other** menstrual phase)   \| Late follicular:  ↔ Thromboxane B2 levels  **platelet reactivity induced by:**  ↔ ADP  ↔ Collagen \| Midluteal:  ↑ Thromboxane B2 levels  **platelet reactivity induced by:**  ↔ ADP  ↔ Collagen \| \| --- \| --- \|   **Post-exercise:** (arrows indicate **vs. pre**-exercise values)   \| Late follicular:  ↑ Thromboxane B2 levels  **platelet reactivity induced by:**  ↔ ADP  ↑ Collagen \| Midluteal:  ↔ Thromboxane B2 levels  **platelet reactivity induced by:**  ↔ ADP  ↑ Collagen \| \| --- \| --- \| | (Ersöz et al., 2002)  (n=16) |

Values are Mean ± SD unless stated otherwise.

Table 2. The effect of different training protocols on biomarkers of thrombogenicity.

| **Subject characteristics** | **Exercise mode // duration // intensity** | **Biochemical markers of blood homeostasis: Post vs. pre**- **exercise training** | **Reference** |
| --- | --- | --- | --- |
| **Males only**  Training (n=26)  Age: 30 to 49 yr.  VO2_max_ (ml kg min^-1^): 38.4 before  VO2_max_ (ml kg min^-1^): n.s . after  Control (n=27)  Age: 30 to 49 yr.  VO2_max_ (ml kg min^-1^): 36.1 before  VO2_max_ (ml kg min^-1^): n.s. after | Brisk walking + slow jogging // 3 months - 45 to 60 min, 5 x week // moderate/low-intensity | (arrows indicate **vs. pre** training resting platelet reactivity)   \| **Training**  ↓ ADP induced platelet aggregation \| **Control**  ↔ ADP induced platelet aggregation \| \| --- \| --- \| | (Rauramaa et al., 1986) (n=53) |
| **Males only**  Control (n=12)  Age: 21.7 ± 2.2 yr.  VO2_max_ (ml kg min^-1^): ~34 before  VO2_max_ (ml kg min^-1^): ~44 after  Training (n=11)  Age: 21.0 ± 2.3 yr.  VO2_max_ (ml kg min^-1^): ~34.5 before  VO2_max_ (ml kg min^-1^): n.s. after | Bike-ergometer // 2 months – 30min, 5 x week // moderate-intensity | (arrows indicate **vs. pre** training platelet reactivity after acute maximal exercise)   \| **Training**  ↓ ADP induced platelet aggregation \| **Control**  ↔ ADP induced platelet aggregation \| \| --- \| --- \| | (Wang et al., 1995)  (n=21) |
| **Females only**  Training (n=8)  Age: 22 ± 1.3 yr.  VO2_max_ (ml kg min^-1^): ~26 before  VO2_max_ (ml kg min^-1^): ~36 after  Control (n=8)  Age: 21.3 ± 0.8 yr.  VO2_max_ (ml kg min^-1^): ~25 before  VO2_max_ (ml kg min^-1^): n.s. after | Bike-ergometer // 2 menstrual cycles – 30min, 5 x week // moderate-intensity | (arrows indicate **vs. pre** training platelet reactivity after acute maximal exercise)   \| **Training**  ↓ ADP induced platelet aggregation \| **Control**  ↔ ADP induced platelet aggregation \| \| --- \| --- \| | (Wang et al., 1997)  (n=16) |
| **Males and Females (grouped)**  Exercise (7M/6F)  Age: 32.1 ± 6.4 yr.  VO2_max_ (ml kg min^-1^): 42.9 ± 2.3 before  VO2_max_ (ml kg min^-1^): 47.8 ± 2.7 after  Control (6M/6F)  Age: 33.4 ± 5.4 yr.  VO2_max_ (ml kg min^-1^): 43.5 ± 0.4 before  VO2_max_ (ml kg min^-1^): 44.0 ± 2.8 after | Bike-ergometer // 3 months - 30 min, 3 x week // moderate-intensity | (arrows indicate **vs. pre** training plasma markers at rest and after acute maximal exercise)   \| **Training**  ↔ APTT  ↔ Factor VIII antigen \| **Control**  ↔ APTT  ↔ Factor VIII antigen \| \| --- \| --- \| | (El-Sayed et al., 1995)  (n=25) |
| **Males only**  Age: 26 ± 3.6 yr. (n=13)  VO2_max_ (ml kg min^-1^): 48.4 ± 5.8 before  VO2_max_ (ml kg min^-1^): 56.5 ± 6.1 after  Age: 40 ± 3.6 yr. (n=13)  VO2_max_ (ml kg min^-1^): 41.2 ± 6.1 before  VO2_max_ (ml kg min^-1^): 47.2 ± 6.9 after  Age: 54 ± 3.6 yr. (n=13)  VO2_max_ (ml kg min^-1^): 31.2 ± 4.0 before  VO2_max_ (ml kg min^-1^): 36.6 ± 5.1 after | Bike-ergometer // 3 months - 60 min, 2 x week // moderate-intensity | (arrows indicate **vs. pre** training plasma markers immediately after acute maximal exercise)   \| **Age 20-30:**  ↑ t-PA Activator  ↑ t-PA Antigen  ↑ Factor VIII:C  ↓ APTT  ↑ vWF Activity  ↔ D-dimer \| **Age 35-45**:  ↑ t-PA Activator  ↑ t-PA Antigen  ↑ Factor VIII:C  ↓ APTT  ↑ vWF Activity  ↔ D-dimer \| **Age 50-60:**  ↑ t-PA Activator  ↑ t-PA Antigen  ↑ Factor VIII:C  ↓ APTT  ↔ vWF Activity  ↔ D-dimer \| \| --- \| --- \| --- \| | (van den Burg et al., 2000)  (n=39) |
| **Females only**  Pre-menopausal (n=13)  Age: 49.1 ± 1.4 yr.  VO2_max_ (ml kg min^-1^): 31.5 ± 2.2 before  VO2_max_ (ml kg min^-1^): 34.8 ± 3.2 after  Post-menopausal (n=14)  Age: 53.7 ± 2.2 yr.  VO2_max_ (ml kg min^-1^): 30.4 ± 3.4 before  VO2_max_ (ml kg min^-1^): 33.5 ± 4.1 after | Bike-ergometer // 3 months - 1h, 3 x week // high-intensity | (arrows indicate **vs. pre** training resting platelet reactivity)   \| **Pre**-menopausal  **platelet reactivity induced by:**  ← TRAP-6  ↓ ADP  ↓ Collagen \| **Post**-menopausal  **platelet reactivity induced by:**  ← TRAP-6  ↔ ADP  ↔ Collagen \| \| --- \| --- \| | (Lundberg Slingsby et al., 2017) (n=27) |

Values are Mean ± SD unless stated otherwise.

Table 3. Influence of an acute exercise bout on biomarkers of thrombogenicity in patient groups compared to healthy controls.

| **Subject characteristics** | **Exercise mode // duration // intensity** | **Biochemical markers of blood homeostasis: Post** **vs. pre acute** **exercise** | **Reference** |
| --- | --- | --- | --- |
| **Males and Females** **(grouped)**  Control 11 Males & 9 Females  Age: 63 ± 10 yr.  Ankle brachial pressure  index: 1.14 ± 0.10  Patients with intermittent claudication***** 16 Males & 4 Females  Age: 68 ± 8 yr.  Ankle brachial pressure  index: 0.62 ± 0.13 | Treadmill (speed 3.5 km h^-1^, incline 5°) // maximum walking distance or corresponding average for control (3min, 20sec)  * medicated with 75mg aspirin; 40 mg of simvastatin or pravastatin and 10 mg of atorvastin | (arrows indicate **patient vs. control** plasma markers)   \| **Before:**  ↑ soluble P-selectin  ↔ vWF  **Platelet reactivity induced by:**  ↓ TRAP  ↓ COX \| **Immediately post:**  ↑ soluble P-selectin  ↔ vWF  **Platelet reactivity induced by:**  ↓ TRAP  ↓ COX \| **1 hour post:**  ↑ soluble P-selectin  ↔ vWF \| \| --- \| --- \| --- \| | (Collins et al., 2006)  (n=40) |
| **Males and Females (grouped)**  Control 15 Males & 3 Females  Age: 33.9 ± 6.2 yr.  BMI (kg m^-2^): 23.54 ± 2.66  Hypertensives# 22 Males & 4 Females  Age: 38.1 ± 6 yr.  BMI (kg m^-2^): 28.35 ± 2.57  Coronary artery disease patients* 13 Males & 3 Females  Age: 40.9 ± 3.9 yr.  BMI (kg m^-2^): 29.07 ± 4.26 | Treadmill exercise test by modified protocol by Bruce (increased gradually from  2.7 km/h (1.7 mph) and 10% grade incline every three minutes)  // 5 stages (15 min)  # not using antihypertensive medication  * Medication of CAD group were paused for at least one week. The use of aspirin, non-steroid anti-inflammatory drugs or other drugs that interfere with platelet function was prohibited for two weeks prior to experiment. | **Pre-exercise:** (one arrow indicates **vs. Control** resting platelet reactivity)   \|  \| Hypertensive:  ↔ Thromboxane B2 levels  ↑ Epinephrine  ↑ Norepinephrine \| Coronary artery disease:  ↔ Thromboxane B2 levels  ↑ Epinephrine  ↑ Norepinephrine \| \| --- \| --- \| --- \|   **15 min treadmill:** (arrows indicate **vs. inherent** pre-exercise platelet reactivity and plasma markers; double arrow **vs. Control** in addition)   \| Control:  ↔ Thromboxane B2  ↑ Epinephrine  ↑ Norepinephrine  **Platelet reactivity induced by:**  ↓ Collagen  ↓ ADP \| Hypertensive:  ↔ Thromboxane B2 levels  ↑↑ Epinephrine  ↑↑ Norepinephrine  **Platelet reactivity induced by:**  ↓ Collagen  ↓ ADP \| Coronary artery disease:  ↔ Thromboxane B2 levels  ↑↑ Epinephrine  ↑↑ Norepinephrine  **Platelet reactivity induced by:**  ↓ Collagen  ↓ ADP \| \| --- \| --- \| --- \|   **10 min post:** (arrows indicate **vs. inherent** resting platelet reactivity and plasma markers; double arrow **vs. Control** in addition)   \| Control  ↔ Thromboxane B2 levels  ↑ Epinephrine  ↑ Norepinephrine  **Platelet reactivity induced by:**  ↓ Collagen  ↓ ADP \| Hypertensives**:**  ↔ Thromboxane B2 levels  ↑↑ Epinephrine  ↑ ↑ Norepinephrine  **Platelet reactivity induced by:**  ↔ Collagen  ↔ ADP \| Coronary artery disease:  ↔ Thromboxane B2 levels  ↑↑ Epinephrine  ↑↑ Norepinephrine  **Platelet reactivity induced by:**  ↔ Collagen  ↔ ADP \| \| --- \| --- \| --- \| | (Petidis et al., 2008)  (n=60) |
| **Males and Females (grouped)**  Control* 17 Males & 14 Females  Age: 35.2 ± 9.4 yr.  VO2_peak_ (ml kg min^-1^): 37.84 ± 8.66  Hypertensives* 12 Males & 7 Females  Age: 46.8 ± 7.5 yr.  VO2_peak_ (ml kg min^-1^): 31.04 ± 10.55 | Treadmill // 20 min // Moderate intensity (65–70% VO2peak)  *no medication 1 week prior to  exercise | (arrows indicate **Hypertensives vs. control** plasma markers)   \| **Before:**  ↔ Epinephrine  ↔ Norepinephrine  **Platelet reactivity by:**  ↔ Unstimulated  ↔ ADP \| **Immediately post:**  ↔ Epinephrine  ↔ Norepinephrine  **Platelet reactivity by:**  ↔ Unstimulated  ↔ ADP \| **25 min post:**  ↔ Epinephrine  ↔ Norepinephrine  **Platelet reactivity by:**  ↑ Unstimulated  ↔ ADP \| \| --- \| --- \| --- \| | (Hong et al., 2009)  (n=50) |
| **Males and Females (grouped)**  Control 16 Males & 4 Females  Age: 51 ± 7 yr.  Coronary artery disease patients* 46 Males & 16 Females  Age: 54 ± 8 yr. | Treadmill exercise test by modified protocol by Bruce (increased gradually from 2.7 km/h (1.7 mph) and 10% grade incline every three minutes) // 7.2 ± 1.3 min in patient group // till onset of symptoms  * Experiment 6 to 13 months (mean 8) after percutaneous revascularization | (arrows indicate **patient vs. control** plasma markers)   \| **Pre**:  **Platelet reactivity by:**  NA collagen/epinephrine  NA collagen/ADP \| **1 min post:**  **Platelet reactivity by:**  ↑ collagen/epinephrine  ↔ collagen/ADP \| \| --- \| --- \| | (Pamukcu et al., 2005)  (n=82) |
| **Males only**  **Acute coronary syndrome patients undergoing cardiac rehabilitation**  Moderate-intensity continuous training (MICT) (n=42)  Age: 61.7 ± 9.8 yr.  VO2_max_ (ml kg min^-1^): 23.2 ± 5.4  high-intensity interval training (HIIT) + MICT (n=40)  Age: 60.0 ± 9.4 yr.  VO2_max_ (ml kg min^-1^): 23.1 ± 5.0 | Bike-ergometer // 3 months – 20-60 min, 4 x week //  MICT: 4x/week moderate intensity training  HIIT + MICT: 2x/week high-intensity training (>90% HR_peak_) + 2x/week moderate intensity training  Exercise test: Bike-ergometer // incremental increase (1 min step-protocol) // Maximal intensity | **Pre training** (arrows indicate **changes in HIIT + MICT** **vs. MICT** training platelet reactivity before (rest) and after acute maximal exercise)   \| **Rest**  ↔ Soluble P-selectin  **Platelet reactivity by:**  ↔ TRAP-6 \| **Immediately post:**  ↔ Soluble P-selectin  **Platelet reactivity by:**  ↔ TRAP-6 \| \| --- \| --- \|   **6-week training period:** (arrows indicate **vs. MICT** resting reactivity)   \| **Rest**  ↔ Soluble P-selectin  **Platelet reactivity by:**  ↓ TRAP-6 \| **Immediately post:**  ↔ Soluble P-selectin  **Platelet reactivity by:**  ↓ TRAP-6 \| \| --- \| --- \|   **12-week training period:** (arrows indicate **vs. MICT** resting reactivity)   \| **Rest**  ↔ Soluble P-selectin  **Platelet reactivity by:**  ↓ TRAP-6 \| **Immediately post:**  ↔ Soluble P-selectin  **Platelet reactivity by:**  ↓ TRAP-6 \| \| --- \| --- \| | (Heber et al., 2020)  (n=82) |

Values are Mean ± SD unless stated otherwise.

Collins, P., Ford, I., Ball, D., Macaulay, E., Greaves, M., and Brittenden, J. (2006). A Preliminary Study on the Effects of Exercising to Maximum Walking Distance on Platelet and Endothelial Function in Patients with Intermittent Claudication. *European Journal of Vascular and Endovascular Surgery* 31**,** 266-273.

Davies, N.A., Llwyd, O., Brugniaux, J.V., Davies, G.R., Marley, C.J., Hodson, D., Lawrence, M.J., D'silva, L.A., Morris, R.H., Hawkins, K., Williams, P.R., Bailey, D.M., and Evans, P.A. (2016). Effects of exercise intensity on clot microstructure and mechanical properties in healthy individuals. *Thromb Res.* 143:130-6.**,** 10.1016/j.thromres.2016.1005.1018. Epub 2016 May 1020.

El-Sayed, M.S., Lin, X., and Rattu, A.J. (1995). Blood coagulation and fibrinolysis at rest and in response to maximal exercise before and after a physical conditioning programme. *Blood Coagul Fibrinolysis* 6**,** 747-752.

Ersöz, G., Zergeroğlu, A.M., and Yakaryilmaz, A. (2002). The effect of submaximal exercise on platelet aggregation during late follicular and midluteal phases in women. *Thromb Res* 108**,** 147-150.

Gunga, H.C., Kirsch, K., Beneke, R., Böning, D., Hopfenmüller, W., Leithäuser, R., Hütler, M., and Röcker, L. (2002). Markers of coagulation, fibrinolysis and angiogenesis after strenuous short-term exercise (Wingate-test) in male subjects of varying fitness levels. *Int J Sports Med* 23**,** 495-499.

Handa, K., Terao, Y., Mori, T., Tanaka, H., Kiyonaga, A., Matsunaga, A., Sasaki, J., Shindo, M., and Arakawa, K. (1992). Different coagulability and fibrinolytic activity during exercise depending on exercise intensities. *Thromb Res* 66**,** 613-616.

Heber, S., Fischer, B., Sallaberger-Lehner, M., Hausharter, M., Ocenasek, H., Gleiss, A., Fischer, M.J.M., Pokan, R., Assinger, A., and Volf, I. (2020). Effects of high-intensity interval training on platelet function in cardiac rehabilitation: a randomised controlled trial. *Heart* 106**,** 69-79.

Hegde, S.S., Goldfarb, A.H., and Hegde, S. (2001). Clotting and fibrinolytic activity change during the 1 h after a submaximal run. *Med Sci Sports Exerc* 33**,** 887-892.

Hong, S., Adler, K.A., Von Känel, R., Nordberg, J., Ziegler, M.G., and Mills, P.J. (2009). Prolonged platelet activation in individuals with elevated blood pressure in response to a moderate exercise challenge. *Psychophysiology* 46**,** 276-284.

Lundberg Slingsby, M.H., Gliemann, L., Thrane, M., Rytter, N., Egelund, J., Chan, M.V., Armstrong, P.C., Warner, T.D., and Hellsten, Y. (2018). Platelet responses to pharmacological and physiological interventions in middle-aged men with different habitual physical activity levels. *Acta Physiol (Oxf).* 223**,** e13028. doi: 13010.11111/apha.13028. Epub 12018 Jan 13019.

Lundberg Slingsby, M.H., Nyberg, M., Egelund, J., Mandrup, C.M., Frikke-Schmidt, R., Kirkby, N.S., and Hellsten, Y. (2017). Aerobic exercise training lowers platelet reactivity and improves platelet sensitivity to prostacyclin in pre- and postmenopausal women. *J Thromb Haemost* 15**,** 2419-2431.

Menzel, K., and Hilberg, T. (2011). Blood coagulation and fibrinolysis in healthy, untrained subjects: effects of different exercise intensities controlled by individual anaerobic threshold. *Eur J Appl Physiol* 111**,** 253-260.

Pamukcu, B., Oflaz, H., Acar, R.D., Umman, S., Koylan, N., Umman, B., and Nisanci, Y. (2005). The Role of Exercise on Platelet Aggregation in Patients with Stable Coronary Artery Disease: Exercise Induces Aspirin Resistant Platelet Activation. *Journal of Thrombosis and Thrombolysis* 20**,** 17-22.

Petidis, K., Douma, S., Doumas, M., Basagiannis, I., Vogiatzis, K., and Zamboulis, C. (2008). The interaction of vasoactive substances during exercise modulates platelet aggregation in hypertension and coronary artery disease. *BMC Cardiovasc Disord* 8**,** 11.

Rauramaa, R., Salonen, J.T., Seppänen, K., Salonen, R., Venäläinen, J.M., Ihanainen, M., and Rissanen, V. (1986). Inhibition of platelet aggregability by moderate-intensity physical exercise: a randomized clinical trial in overweight men. *Circulation* 74**,** 939-944.

Röcker, L., Drygas, W.K., and Heyduck, B. (1986). Blood platelet activation and increase in thrombin activity following a marathon race. *Eur J Appl Physiol Occup Physiol* 55**,** 374-380.

Van Den Burg, P.J., Hospers, J.E., Mosterd, W.L., Bouma, B.N., and Huisveld, I.A. (2000). Aging, physical conditioning, and exercise-induced changes in hemostatic factors and reaction products. *J Appl Physiol (1985)* 88**,** 1558-1564.

Wang, J.S., Jen, C.J., and Chen, H.I. (1995). Effects of exercise training and deconditioning on platelet function in men. *Arterioscler Thromb Vasc Biol* 15**,** 1668-1674.

Wang, J.S., Jen, C.J., and Chen, H.I. (1997). Effects of chronic exercise and deconditioning on platelet function in women. *J Appl Physiol (1985)* 83**,** 2080-2085.
